# Changes to Visual Parameters Following Virtual Reality Gameplay

**DOI:** 10.22599/bioj.257

**Published:** 2022-06-27

**Authors:** Sanjog Banstola, Kerry Hanna, Anna O’Connor

**Affiliations:** 1 University of Liverpool, GB

**Keywords:** Virtual Reality, Gameplay, Visual Changes, Vision

## Abstract

**Introduction:**

Virtual reality (VR) gameplay is popular with a range of games and educational resources available. However, it puts high demands on the visual system. Current evidence shows conflicting impacts on visual parameters. Therefore, this study explores the changes to vision following VR gameplay.

**Methods:**

The study was conducted at the School of Health Sciences, University of Liverpool. All participants had binocular vision with good visual acuity and no manifest strabismus. Participants were assessed before and after playing 15 minutes of the VR game Beat Saber, which incorporated convergence and divergence movements. Clinical assessments including near point of convergence (NPC) and near point of accommodation (NPA) using the RAF rule; accommodative convergence to accommodation (AC/A) ratio; motor fusion using the prism fusion range (at 33cm), accommodation facility using +2.00/–2.00DS flipper lenses, and stereoacuity using the Frisby stereo test were assessed before and after playing.

**Results:**

Seventy-eight participants (19–25 years old) were included in the study, with 16 males and 41 females respectively. The breakpoint of convergence reduced by 0.5 cm (p = 0.001). The binocular accommodative facility improved by 2 cycles per minute (cpm); p = 0.004. The mean, near horizontal prism fusion range (PFR) base break and recovery points both worsened by of 5.0 dioptres (p = 0.003), whereas the mean near horizontal PFR base in recovery point improved by of 4.0 dioptres (p = 0.003).

**Discussion:**

The study validated previous findings as VR gameplay over-exercised and fatigued convergence muscles, but to a small degree. The VR experience improved the participants’ ability to change focus quickly and improve accommodation, as well as the divergence function of the eye. However, as the participants were retested directly after the VR gameplay, the findings were limited to short term effects on vision.

## Introduction

The use of virtual reality (VR) is becoming more widespread, with the potential of the technology ranging from entertainment and education to medical interventions (Izard et al. 2018; Izard et al. 2017). VR is a computer-generated simulated experience that is traditionally created by a head-mounted display with a small screen for viewing (Martin 2017). It typically uses auditory and video feedback to give the user a sense of control over their virtual environment. The technology can be distinguished into immersive and non-immersive experiences. For the purpose of this research immersive VR technology is used, where the user is able to interact with the environment by moving around within it.

Given the visual demands of VR, there is an increasing body of evidence evaluating the impact on the visual system, with studies showing impacts on monocular and binocular vision, as well as eye alignment. Positive impacts have been shown in terms of eye alignment, where VR gameplay has shown the potential to treat intermittent exotropia by improving the patients’ exotropia from 21.44 (±13.25 SD) to 7.2 (±8.54 SD) prism dioptres (Δ) on the prism cover test (Li et al. 2019). Regarding monocular vision, it has shown to improve visual acuity in the amblyopic eye by improving the best correct visual acuity from a mean value of 0.58 (±0.35) logMAR to 0.43 (±0.38) logMAR (p < 0.01) (Žiak et al. 2017). However, the technology has shown to cause negative effects on monocular vision, as a study reported that it significantly worsened the near point of accommodation (NPA) of its users by 1.5cm (p < 0.01) in both of their dominant and non-dominant eyes (Yoon et al. 2020).

With regards to binocular vision, VR gameplay has shown conflicting effects on accommodation by improving binocular accommodative facility amongst users with normal binocular function, where the eyes were not exposed to fatigue, whilst also worsening the accommodative-convergence/accommodation (AC/A) ratio in others due to continual change in depth leading to an exo-shift of the horizontal phoria (Munsamy et al. 2020; Mohamed Elias et al. 2019). Similarly, the VR technology has been shown to impact the near point of convergence (NPC) in convergence insufficiency (CI) patients (Irving et al. 2017), where the training methods require individuals to repeatedly converge and re-diverge. This has resulted in further improvements of one’s binocular vision (the recovery point in near prism fusion range from 27.1 (+ 14.2) Δ to 34.9 (+ 13.5) Δ). Therefore, the findings from this study suggest that VR could be useful in managing patients with CI, namely those with neurological and neurodegenerative conditions.

However, it has also worsened NPC by 2 cm (p < 0.01) in healthy users with good binocular vision (Yoon et al. 2020; M. Y. Boon 2017). Furthermore, the same pattern has been seen with stereopsis as dichoptic training in patients with amblyopia (viewing of separate fields by each eye) has shown improved stereoacuity from 263.3 (±135.1) to 176.7 (±152.4) seconds of arc (p < 0.01) (Žiak et al. 2017), whilst a different study on healthy participants has shown no improvement of stereopsis from VR technology (Yoon et al. 2020). Therefore, it is likely that where patients have deficient visual parameters at baseline, VR can support improvement in clinical measures, whilst those with normal levels of vision appear to be unchanged after VR use.

Besides vision function tests, previous research has found conflicting vergence and accommodation relationship associated with virtual reality (Iskander et al. 2019). Use of VR can lead to physical visual stress by affecting visual acuity and causing greater symptoms such as headache, diplopia, blurred vision, sore eyes and eyestrain for the user (Yoon et al. 2020; Tychsen and Foeller 2020). The conflict arises due to the inability of the eyes to focus on the projection screen whilst converging on the virtual object. Furthermore, a study has shown strong correlation between symptomatic patients and poor vision function tests, such as increased exophoria and reduced AC/A ratio, following VR exposure (Morse and Jiang 1999).

With the true effect of virtual reality still needing more clarification, with previous studies showing improvements of some visual parameters whilst worsening others, finding how this technology affects vision remains an objective that needs to be addressed. Hence, this study will aim to provide greater clarification of the changes to visual function measures that have shown conflicting findings in previous literature (NPC, NPA, accommodative facility, AC/A ratio, stereoacuity, and prism fusion range) by observing the changes to visual parameters before and after 15 minutes of VR gameplay.

## Materials and Methods

### Study Design and Setting

A prospective observational cohort study was conducted using data from a year two undergraduate research project module where eight student-led subgroups collected the data, with close supervision from two university lecturers. Volunteer participants were university students recruited using advertisements on email, posters, and social media between February and March 2019. The study involved collecting baseline information to screen for inclusion in the study, followed by a visual assessment before and after 15 minutes of virtual reality gameplay, observing any changes to the visual function parameters. Fifteen minutes was the chosen duration, as this was both clinically feasible within the constraints of this research study and was informed by previous literature, which reported significant results to vision after only 10 minutes of VR gameplay (Szpak et al. 2020). The participants played Beat Saber, a VR game where the user is required to slash blocks as they come towards them (Games 2021). As a result, the game required the person to make binocular convergence and redivergence movements.

### Participants

Participants of the study were included following a screening process. The inclusion criteria consisted of:

- Aged between 18 and 40 years old to exclude cases of presbyopia (Fricke et al. 2018)
- Presence of stereoacuity and no manifest strabismus on cover test assessment.
- Good and equal visual acuity (VA), defined as a difference of VA less than 0.1 logMAR between the two eyes. Each eye must also achieve 0.2 logMAR.
- Had mental capacity and the ability to consent.

### Ethical Approval

All participants of the study were able to provide a written informed consent after reading the patient information sheet before enrolling onto the experiment. The study was approved by the Research Ethics Approval Committee of the University of Liverpool.

### Data Collection

The study gathered data from eight subgroups, each of which aimed to enroll 10 participants. Demographic data collected were age, gender, and past ocular history, whilst the clinical data included visual acuity, cover test, and interpupillary distance (IPD). As part of the basic visual assessment before and after VR gameplay, based on existing scientific evidence, each subgroup chose at least three appropriate visual function parameters to test on their volunteers. The student assessors were required to select at least three outcome measures to meet the needs of the undergraduate module assessment. The module teaching staff, co-author (KH), closely supported the students to ensure relevant outcome measures were selected for this study, supported by previous evidence obtained earlier in the module through literature searching. However, as each group chose different visual function tests, it was expected that each parameter would have missing values at the analysis stage. Each subgroup collected the data in a Microsoft Excel spreadsheet, from which a central database containing all the screening and experimental data was later formed for the current study analysis.

### Outcome measures

Changes in the visual parameters before and after VR gameplay included: NPC and NPA at break and recovery points using the RAF rule (Adler 2004); near horizontal PFR using handheld prism bars, with blur, break, and recovery point measured for base in (BI) and base out (BO); binocular accommodative facility using +2.00/–2.00DS flipper lenses; AC/A ratio (calculated using the gradient method); and the Frisby stereoacuity test (Frisby 2014). All measures were chosen based on previous evidence outlining impact from VR use; however, the near horizontal prism fusion range has limited previous evidence but was included to explore the impact and provide greater understanding.

### Data Analysis

After the formation of the central database, the spreadsheet was exported and analysed using Statistical Package for the Social Sciences (SPSS) version 25 (IBM 2021). Analysis showed the data were not normally distributed (Shapiro-Wilk), thus descriptive analysis was conducted using medians and interquartile ranges and illustrated with the number and percentage of the demographical and screening data. Furthermore, the visual parameters were analysed using a matched non-parametric test, the Wilcoxon signed-rank test, exploring any significant changes due to VR gameplay. Additionally, the Bonferroni correction method was used to counteract the possibility of false significance due to multiple comparisons of the data (Curtin and Schulz 1998). As 14 comparisons were planned to be carried out (thus producing the calculation 0.05/14 = 0.004), a p-value of less than or equal to 0.004 was considered statistically significant for the purposes of this study.

## Results

### Demographics and Screening

A total of 79 participants were recruited in the study and, after the application of inclusion criteria and accounting for one subgroup failing to recruit 10 participants, 78 participants were included in the study. In addition, various aspects of participant’s demographical and screening data were missing (Table 1); however, enough data was recorded to be included in the analysis. Furthermore, the screening measures between those with missing demographic data (gender and age) and those without missing data produced no statistically significant findings, therefore allowing for further analysis of the experimental measures (VA right eye p = 0.99, VA left eye p = 0.99, CT near p = 0.80, CT distance p = 0.091). The median age of the participants was 20 years (IQR 1.0 years) and there was a considerably greater number of females compared to male participants (41 vs 16 participants, respectively). Of the 38 participants that had their past ocular history recorded, the majority of the individuals did not have any history to disclose, whilst four reported a manifest strabismus (n = 1) and a convergence insufficiency (n = 1), with the remaining two histories being unrecorded. It is important to note that three participants (n = 1 CI and n = 2 unknown) had recovered from their presenting complaints and demonstrated binocular vision with good and equal visual acuity at the time of the experiment, and thus were included in the study.

**Table 1:** Outline of the participant demographics and screening data.


		AFTER EXCLUSION* N = 78	MISSING VALUES, N (%)

**Age in year (median, IQR)**		20.0 (1.0)	22 (27.5)

**Gender, N (%)**	**Male**	16 (20.5)	22 (27.5)

	**Female**	41 (52.6)	

**Past Ocular History, N (%)**	**Present**	4 (51.3)	40 (51.2)

	**Absent**	34 (43.6)	

**Visual Acuity, logMAR (median, IQR)**	**Right eye**	0.0 (0.2)	1 (1.3)

	**Left eye**	0.0 (0.1)	

**Near Cover Test, N (%)**	**Esophoria**	9 (11.5)	1 (1.3)

	**Exophoria**	39 (50.0)	

	**Vertical Phoria**	1 (1.3)	

	**NAD**	29 (37.2)	

**Distance Cover Test, N (%)**	**Esophoria**	3 (3.8)	21 (26.3)

	**Exophoria**	14 (17.9)	

	**Vertical Phoria**	1 (1.3)	

	**NAD**	40 (51.3)	

**IPD, mm (median, IQR)**		60 (4.5)	33 (41.3)


* After exclusion accounts for data after exclusion criteria applied and missing values removed. N = number of participants, IQR = interquartile range, logMAR = the logarithm of the Minimum Angle of Resolution, NAD = no abnormality detected.

In terms of the screening data, the cohort showed good visual acuity with median values of 0.0 logMAR (IQR 0.1 and 0.2 logMAR) across both eyes. The near and distance cover test confirmed one individual to have a manifest strabismus and they were excluded from the study. The median IPD was 60mm (IQR 4.5mm), although 33 patients did not have this data recorded.

### Experimental results

One variation between groups was the number of repetitions of the clinical measurements made, but for analysis purposes the first attempt is used to ensure there is no fatigue effect. The changes to visual parameters before and after VR gameplay can be found in Table 2. The NPC break and recovery values worsened by 0.5cm, but only the break of convergence was statistically significant (p = 0.001). The NPA was only carried out by one subgroup (10 individuals), and showed no statistically significant changes before and after VR gameplay.

**Table 2:** Summary of the changes to visual parameters before and after VR gameplay.


VISUAL PARAMETERS		BEFORE VR GAMEPLAY	AFTER VR GAMEPLAY**	P-VALUE	VALUES INCLUDED, N (%)

**NPC, cm** **(median, IQR)**	**Break**	6.0 (1.0)	6.5 (3.0)	0.001*	68 (87.2)

	**Recovery**	6.5 (2.8)	7.0 (3.0)	0.526	20 (25.6)

**NPA, cm** **(median, IQR)**	**Break**	8.0 (3.0)	8.0 (7.0)	0.026	10 (12.8)

**Accommodative Facility, cpm (median, IQR)**	**Both eyes**	11.0 (5.0)	13.0 (5.0)	0.004*	58 (74.3)

	**Right eye**	12.5 (8.5)	9.5 (15.25)	0.888	10 (12.8)

	**Left eye**	15.0 (8.5)	12.0 (10.5)	0.482	10 (12.8)

**AC/A ratio (median, IQR)**		2.0 (2.0)	1.5 (2.3)	0.389	58 (74.3)

**Frisby, log10secs of arc** **(median, IQR)**		1.74 (1.2)	1.74 (1.2)	0.317	20 (25.6)


* Significant values after Bonferroni correction applied. ** After 15 minutes of playing Beat Saber using a VR headset. N = number of participants, IQR = interquartile range, cpm = cycles per minute. p-value calculated using Wilcoxon signed rank test followed by Bonferroni correction resulting in a statistically significant level of p-value < 0.004.

Binocular accommodative facility, recorded in 58 out of 78 participants, improved from 11.0 to 13.0 cpm (p = 0.004). However, one subgroup (n = 10) measured accommodative facility monocularly and revealed worsening of 3 cpm in each eye, but the findings were statistically insignificant (p = 0.888 right eye and p = 0.482 left eye). The AC/A ratio and stereoacuity measures were not statistically different after VR gameplay (p = 0.389 and p = 0.317, respectively).

For the BO near horizontal PFR (Table 3), the median blur point worsened from 19.0Δ to 16.0Δ in 48 participants but was not statistically significant (p = 0.008). The median break point (observation of double vision) also worsened from 35.0Δ to 30.0Δ in 68 participants of the study and was statistically significant (p = 0.003). The BO near horizontal PFR recovery point, which was recorded in 48 out of 78 participants of the study, also worsened from 30.0Δ to 25.0Δ (p = 0.003).

**Table 3:** The changes to near horizontal PFR before and after VR gameplay.


NEAR HORIZONTAL PFR, DIOPTRES (MEDIAN, IQR)		BEFORE VR GAMEPLAY	AFTER VR GAMEPLAY**	P-VALUE	VALUES INCLUDED, N (%)

**Base Out**	**Blur**	19.0 (9.0)	16.0 (11.0)	0.008	48 (61.5)

	**Break**	35.0 (20.0)	30.0 (15.0)	0.003*	68 (87.2)

	**Recovery**	30.0 (18.8)	25.0 (17.0)	0.003*	48 (61.5)

**Base In**	**Blur**	12.0 (4.0)	13.0 (4.0)	0.006	48 (61.5)

	**Break**	14.0 (6.0)	16.0 (8.0)	0.024	68 (87.2)

	**Recovery**	12.0 (5.5)	16.0 (8.0)	0.003*	48 (61.5)


* Significant values after Bonferroni correction applied. ** After 15 minutes of playing Beat Saber using a VR headset. N = number of participants, IQR = interquartile range. p-value calculated using Wilcoxon signed rank test followed by Bonferroni correction resulting in a statistically significant level of p-value <0.004.

On the other hand, the BI near horizontal PFR showed improvement following VR gameplay. Although the BI blur point improved from a median of 12.0Δ to 13.0Δ in 48 participants of the study, it was not statistically significant (p = 0.006). However, the median BI break point improved from 14.0Δ to 16.0Δ in 68 participants and was statistically significant (p = 0.024). Lastly, the median BI near horizontal PFR recovery point also significantly improved from 12.0Δ to 16.0Δ (p = 0.003) in 48 participants. In fact, the median recovery point was equal to the median break point after VR gameplay (16.0Δ).

## Discussion

The study explored changes to vision following VR gameplay by measuring visual parameters before and after playing 15 minutes of the VR game Beat Saber. The findings showed that the VR game, which primarily utilised binocular convergence and redivergence, could be attributable to a slight worsening of motor components of vision such as breakpoint of convergence. This supported previous findings which also suggested worsening NPC following VR gaming on healthy volunteers (Munsamy et al. 2020). However, when VR gaming is applied to participants with CI for a longer period of time, such as three weeks, it has the capability to improve this function, possibly due to suggestions that neuroplasticity can be altered after repeated exposure, compared to just one exposure of VR gaming (Li et al. 2019). Therefore, the worsening of convergence in this study could have been due to fatigue of the eye muscles after carrying out convergence movements during the game, rather than a long-term effect of VR gaming.

The current study found that VR gameplay worsened other convergence measures such as the near horizontal PFR BO break and recovery points. This was contradictory to a study carried out in participants with CI, where individuals followed up for three weeks after VR gameplay showed improved BO near horizontal PFR (M. Y. Boon 2017). The difference in findings could have been due to the alternative VR game played, in which users exercised convergence more than 50% of the time (Boon et al. 2017), compared to that played in the current study where performance was not quantified. In addition, as the study consisted of 15minutes of VR gaming repeated over three weeks (equating to 103 ± 76 minutes of training in total) compared to just one 15 minutes session in the present study, the participants were exposed to greater convergence training. A further study measuring the effect on visual parameters after VR gameplay also showed the changes that were observed after 10 minutes returned to baseline values by 50 minutes (Szpak et al. 2020), suggesting that duration of gameplay is a key factor in change to vision long-term.

The current study showed improvement of divergence as the BI near horizontal PFR recovery point improved following VR gameplay. The improvement of only divergence could be due to the lower demands on the eye to carry out divergence (relaxation) than convergence (constricting) movements. Hence, the muscles assisting divergence were not over-exercised and were able to recover and refocus quicker. Boon et al. (2017) also investigated the changes of this parameter in CI participants and showed no significant findings even after three weeks of VR gaming. However, the authors suggest this could be due to a ceiling effect in the negative fusional vergence ranges. Further studies exploring changes of these visual parameter on healthy individuals need to be carried out to produce conclusive findings.

The present study suggested some improvement of accommodation measures as the binocular accommodative facility improved after the VR experience, which was consistent to previous findings on participants with normal binocular vision (Munsamy et al. 2020). Having to continually focus on a different target during the game may have helped exercise accommodation. Monocular accommodative facility was not altered significantly. However, there were fewer participants included in this subsample. Similarly, other results such as the recovery point of NPC and near horizontal PFR BO blur point from the current study may have been impeded from a smaller subsample compared to the significant results found using a larger subsample of the participants. Therefore, a recommendation from this research is for future studies to assess the impact on these visual parameters after VR gameplay within a larger cohort for accuracy.

Due to limited evidence exploring the impact of VR gameplay on stereoacuity, stereoacuity was also investigated in this study. The results revealed no changes to this parameter following 15 minutes of VR gameplay. A previous study showed improvement of stereoacuity in adults with amblyopic eyes; however, a contrasting study carried out on healthy individuals had shown no changes to this visual function (Žiak et al. 2017; Yoon et al. 2020). As the current study was also carried out on healthy individuals, it adds to previous findings by showing no effects on stereoacuity following VR headset use.

Although the study has highlighted statistically significant changes to vision following VR gameplay, it is important to consider the clinical significance of these results (Peeters 2016). For the purpose of this study, a worsening of 0.5 cm of NPC, improvement of 2 cpm of binocular accommodative facility, and improvement of 4Δ of near horizontal PFR BI recovery point are all significant results, but it does not reflect a clinical change to vision where the user is able to notice the difference (Abraham et al. 2015; Yekta et al. 2017). Therefore, further studies investigating symptoms and visual complaints from VR users need to be undertaken to assess the clinical consequences of VR gameplay.

The study emphasised the potential harmful impact to vision for children and young adults who typically use the technology more regularly and for a much longer period of time (Vailshery 2018). In addition, for individuals undergoing training to improve a CI, the study suggests advice should be given to reduce the use of this technology. On the other hand, as studies including CI participants with longer exposure time to VR gaming resulted in no improvement of visual parameters (Boon et al. 2017), it highlights the need to find a relationship between duration of VR gameplay and impact on visual function. Therefore, before any guidelines on the use of VR gameplay are established further research with longer exposure and follow-up period should be carried out.

### Strengths

One of the main strengths of this study was the inclusion of multiple visual parameters. Unlike previous studies, which focused on limited number of outcome measures, this study investigated a wide range of theses outcomes ranging across convergence, accommodation, and stereoacuity. This helped ensure all the changes to vision after VR gameplay were explored in the study. Moreover, Bonferroni correction was used to exclude any significant results due to chance. This increased the power of the significant findings of the study and ensured any changes to vision were as a result of VR gameplay.

### Limitations

There were several study limitations that should be noted. The VR headset was not adjustable below an IPD of 60mm. Therefore, approximately half of the participants were wearing an oversized headset. Recent research has shown participants with a mismatch of IPD may experience a lower image quality with less accurate depth perception and greater eye discomfort (P. Hibbard 2020). All of this could have played a confounding role and affected the experimental results, hence reducing the credibility of the findings. This was a restriction caused by the technology and could not be altered manually, highlighting a flaw in the VR technology design.

Another factor that could have affected the results of the study is the use of a different examiner for each subgroup. Despite all the examiners being orthoptic undergraduate students, the inconsistency between the subgroups was not accounted for during the experiment. Furthermore, the use of students rather than professional opticians may have reduced the accuracy of the test results due to their lack of clinical judgment and experience. Future studies comparing accuracy of clinical assessments by a qualified orthoptist could provide clarity on the reliability of these findings.

The timing of the post-VR visual assessment may have impacted on the reliability of the results, where fatigue of the eye following continuous convergence and divergence movements may have skewed the results and masked the long-term effects to vision. Therefore, having a longer follow-up time may have helped observe more accurate changes to vision. In addition, due to the autonomous nature of the student projects, not all variables had complete data for 80 participants. As a result, the study had lots of missing data, which limited the ability to produce significant findings. Future studies should consider having same visual outcomes across all the subgroups to carry out wider comparisons across larger cohorts.

Another aspect to consider for any study that aims to analyze the effects of VR gaming is the game that the study includes. Each game requires different movements of the eye and, as a result, trains different functions of the eye. Hence, when comparing findings of different studies, it is important to acknowledge that effects on vision may vary across different games and the findings may be limited to the particular virtual reality game in question.

## Conclusion

Overall, this study has shown positive and negative changes to vision following just 15 minutes of virtual reality gameplay. The study outlined the improvement of accommodation and divergence functions of the eye, as well as the worsening of convergence following the virtual reality experience. Although the study validated previous research of changes to vision following VR gameplay, the clinical significance of the changes to vision was not established.

Long-term follow up with changes to symptoms and visual complaints from the participants is required to assess the clinical significance. Also, as the study highlighted the impact on healthy individuals, there is a greater need to undertake further research in this population.
